# Natural Killer Cells and Their Implications in Immune Response Diversification in Clinical Pathology and Neoplastic Processes

**DOI:** 10.3390/ijms24097743

**Published:** 2023-04-24

**Authors:** Isabel Legaz, Manuel Muro

**Affiliations:** 1 Department of Legal and Forensic Medicine, Biomedical Research Institute of Murcia (IMIB), Regional Campus of International Excellence “Campus Mare Nostrum”, Faculty of Medicine, University of Murcia (UMU), 30100 Murcia, Spain; 2 Immunology Service, University Clinical Hospital “Virgen de la Arrixaca”—IMIB, 30120 Murcia, Spain

Numerous studies have examined the function of human immune system biomarkers regarding susceptibility, and prognostic, therapeutic, and predictive factors, in various solid and liquid tumors. However, the innate immune mechanisms by which a tumor cell can evade immune checkpoints and immune surveillance are poorly understood.

The innate immune system is a highly dynamic system that includes a variety of cell types, soluble components, ligands, and receptors; moreover, it contains spatial and temporal components, which are crucial because they are linked to the interactions of compounds and toxins with age, gender, and lifestyle choices. From clinical and pathological perspectives, natural killer (NK) cells are crucial to spotting the appearance of cancer. Additionally, these fascinating cells possess a variety of receptors that make it possible to control their natural stimulatory or inhibiting activity. Most of these receptors are involved in numerous omics scenarios (genomics, transcriptomics, epigenomics, interactomics, or proteomics) at the individual level, making cause-and-effect investigations even more challenging in people who have developed tumors. It is essential to characterize, examine, and interpret the receptors and ligands, as well as the mechanisms of action, in various types of tumor, given the considerable potential of NK cell analysis. In order to fully implement this in a clinical context, it is also necessary to build reliable and comprehensive databases that allow users to, among other things, determine the causes of carcinogenesis, metastasis, or recurrence, or predict the time until recurrence occurs. Numerous studies suggest that NK cell analysis has a promising future in several clinical medical science fields, initiating a significant new area for research and development.

In addition to their fundamental role in cancer, these cells and their cell receptors are crucial in the successful and unsuccessful immune response to solid organ and hematopoietic transplants. Finding the underlying causes of illness and death may be facilitated by understanding individual variations in NK cells’ omics relationships.

This new Special Issue of the *International Journal of Molecular Sciences*, entitled “Natural Killer Cells and Their Implications in Immune Response Diversification in Clinical Pathology and Neoplastic Processes”, includes five essential contributions to this interesting field, providing new information, tools, and data on cancer, transplantation, and other pathological processes.

First, Sonkodi et al., 2022 [1] present an interesting study that examines the immunophenotypical characterization of young swimmers from the Hungarian National Swim Team before and after intense acute exercise, with a focus on natural killer cells.

To begin, it is crucial to understand that delayed-onset muscle soreness (DOMS) is characterized by a delayed onset of soreness, muscle stiffness, swelling, loss of force-generating capacity, decreased range of joint motion, and decreased proprioceptive function [2].

According to Cheung et al. [3], several theories have attempted to explain the pathophysiology of DOMS, including lactic acid buildup, muscle spasms, connective tissue damage, muscle damage, and enzyme efflux theory. However, the research teams in question believe no theory can fully explain this puzzling pathophysiology.

To clarify these points, Sonkodi et al., 2022 [1] studied nineteen swimmers (ten females and nine males) who completed an exercise protocol. Sixteen swimmers experienced DOMS. Most of the results of this research supports earlier findings, including the rise in the percentage of CD3+/CD56+ NK cells and CD3+/CD56dim+ NK cells, and the fall in the proportion of CD3+ T cells among lymphocytes, following the exercise protocol. The return of NKT cell activity to pre-exercise levels was consistent with earlier findings backed up by the authors. Interestingly, the percentage of CD3+/CD56+ NKT-like cells did not significantly change in the three swimmers who did not experience delayed-onset muscle soreness.

On the other hand, the proportion of CD3+/CD56+ NKT-like cells in lymphocytes increased in fourteen swimmers and decreased in two swimmers who reported delayed-onset muscle soreness. This study is critical because it is the first to link the uneven control of CD3+/CD56+ NKT-like cells in lymphocytes to delayed-onset muscle soreness.

However, the main limitation of this article, as with other published articles, is that validation of this association must be conducted using a larger sample size to reinforce these data; thus, further studies should be performed in the future.

Second, Greenshpan et al., 2022 [4] discuss altering the promoter’s expression level while maintaining its receptivity to signals from the tumor microenvironment (TME).

New drugs that only target cancerous cells have been actively sought in recent years. Immunotherapy aims to direct the immune system to precisely and selectively attack tumor cells. The use of chimeric antigen receptor T cells is one of the methods currently in use. T and NK, collectively called “cars”, aim to cause cytotoxicity against particular antigen-expressing cells. In order to achieve this, the drug AP1903 dimerizes an inducible caspase 9, which is introduced to the cells, forcing them to undergo apoptosis. T cells with chimeric antigen receptors (CAR) decide to attack cancerous cells rather than healthy tissue. Limiting CAR expression at the tumor site is another safety measure that can be employed. In [4], the Chimeric Antigen Receptor Tumor-Induced Vector (CARTIV) method demonstrated that inducible promoters specific to the tumor microenvironment could limit CAR expression in the tumor. Several suggested modifications could be made to improve the treatment’s effectiveness and CAR T cell therapy.

Here, the expression of the auxiliary molecule and the CAR are driven by the same promoter, highlighting the need to carefully regulate the expression level in order to produce the desired therapeutic effect. The authors demonstrate how the CARTIV method can limit the expression of the transgene in the TME. They explain how to modify the promoter’s expression level while preserving its sensitivity to TME signals, and show that modifying a specific CARTIV promoter is possible using various minimal promoters with different transcriptional intensities. The same TME-responsive portion of many synthetic CARTIV promoters, including miniTK [5], miniCMV [6,7], and YB_TATA [8], have been conjugated using different minimal core promoters.

Depending on the background level, these core promoters exhibit either weak or solid maximal induction. The authors concluded that the desired transgene would be expressed from the tetracycline response element, while the CARTIV miniTK promoter could drive the reverse tetracycline-controlled trans-activator. Withholding doxycycline while the strong TRE promoter drives high expression levels during the “on stat” stage may enable external control of its expression. The combined findings of these authors show that synthetic promoters can be modified robustly and modularly. By achieving an adequate level of expression of CAR or other effector immune effector genes, it is possible to better engineer CAR-T and other immune cells to treat different types of tumors and a greater number of patients.

Using the 14-25-9 mAb, Iraqiet al., 2022 [9] examined membrane-associated PCNA expression in primary bone marrow (BM) mononuclear cells from MM patients, as well as multiple myeloma (MM) cell lines. Multiple myeloma is a type of cancer that damages end organs by growing plasma cells in the bone marrow, causing monoclonal Ig protein to accumulate in the blood and urine, as well as anemia, bone lesions, renal failure, and hypercalcemia [2].

MM lines and primary cells showed variable cell-membrane PCNA following incubation with the 14-25-9 mAb, which correlated with enhanced NK cell antitumor activity. The authors of this study previously stated that NKp44 can recognize PCNA [10]. Interestingly, PCNA, a homotrimer that clamps around nuclear DNA and encourages replication, is a nuclear factor linked to cancer. As a clinical marker for malignancy, the increased expression of PCNA is seen in proliferating cells, including cancer cells. PCNA suppresses the activity of NK cells.

Additionally, the authors showed that PCNA is present on tumor cell surfaces and is drawn to the NK immunological synapse, resolving the mystery of how NKp44 can detect nuclear PCNA in tumor target cells. Intriguingly, there are three different differentially spliced isoforms of NKp44. NKp44 isoform 1 is the only one to express a single cytoplasmic ITIM linked to PCNA’s inhibitory effect on NKp44-mediated function; however, isoforms 2 and 3 lack the ITIM. Therefore, splice-isoform 1, derived from NKp44’s recognition of PCNA on the surface of tumor cells, acts as a novel immune checkpoint that allows tumors to escape immune elimination. In this study, it is suggested that particular tools are required to further analyze PCNA-NKp44 interactions. A monoclonal antibody was recently created that identifies PCNA epitopes exposed on the cell surface. The mAb 14-25-9 successfully prevents PCNA from binding to NKp44. On the other hand, mAb 14-25-9 treatment of different tumors increased NK cell-mediated cytotoxicity in vitro and in PDX models (patient-derived xenograft-bearing NSG mice) [2].

Although their findings offer compelling proof that inhibiting NKp44-PCNA can enhance NK cell-mediated antitumor responses, it is important to note that more research is needed to determine how the Ab 14-25-9 affects NK cells’ responses to various cancer types. The envelope glycoproteins of the West Nile and Dengue flaviviruses, the hemaglutinins of the influenza and Sendai viruses, and the avian Newcastle disease virus are just a few virally induced ligands that NKp44 possesses, in addition to PCNA. By providing pre-clinical evidence in this multiple myeloma study, the authors concluded that PCNA is expressed on the cell surface of MM cells, and that incubation with mAb 14-25-9 increases NK activity against MM cells. The potential for improving the therapeutic outcomes of MM patients by blocking NKp44-PCNA IC, in combination with other established MM therapies, should be explored in clinical trials.

With advancements in the identification of immune response genes, Jimenez-Coll et al., 2023 [11] report changes in the function of various biomarkers and procedures, and in the development of an organ transplant procedure, with regard to the prevention of immunological rejection.

Some of the methods on this list include analyses of immunological epitopes and eplets, the capacity to fix complements, the PIRCHE (Predicted Indirectly Recognizable HLA Epitopes) algorithm, and post-transplant monitoring with promising new biomarkers that outperform traditional serum markers, such as creatine, DGF (delayed graft function), and estimated glomerular filtration rate. Among these new biomarkers, the authors analyze new serological, urine, cellular, genomic, and transcriptomic biomarkers and perform computational prediction, paying particular attention to the analysis of donor-free circulating DNA as an optimal marker of kidney damage [12,13]. Moreover, they discuss possible therapeutic interventions and new delivery strategies aimed at modulating the immune response at several points during transplantation, in order to improve outcomes [14].

Particularly exciting is the ability to define the state of an allograft and its long-term evolution, and prevent the eventual development of an episode of acute or subclinical rejection (when it does not show up immediately and causes unexpected or untreatable graft loss; in many cases, this can be solved via increased immunosuppression and desensitization approaches); even more exciting is the ability to prevent chronic rejection, an event that leads to the progressive loss of the graft. New designs and biomarkers will be available in the immediate future as ongoing clinical trials and molecular signatures complete their final lead-in periods.

Legaz et al. [15] analyzed the influence of Killer Cell Immunoglobulin-like Receptors (KIR) and Human Leucocyte Antigen C (HLA-C), and observed an increase in the risk of long-term chronic liver graft rejection. In this study, recipients with HCV infection comprised 30.2% of the group, while those with HBV infection made up 9.9%. In 6.8% of all patients, CR occurred. In this study, the authors examined the effects of KIRs and HLA-I ligands on long-term liver graft outcomes across all transplants, as well as in chronic (CR) and non-chronic rejection (NCR) groups study. Except for KIR2DS5, which was significantly more prevalent in liver recipients for healthy donors, they found no significant differences in KIR gene distribution between patients and healthy donors.

Additionally, the impact of every KIR gene on CR was assessed. KIR2DL2 markedly improved CR in NCR patients. Within the aKIRs, KIR2DS2 and KIR2DL2 shared a strong relationship, and KIR2DS3 significantly increased the likelihood that patients would experience CR episodes as opposed to NCR (*p* = 0.013 and *p* = 0.038, respectively). All of these associations were verified via multivariable logistic regression analysis, which supported the correlation between liver transplants performed with 0 KIR gene mismatches and those performed with one or more mismatches concerning patients without MM. This was achieved using the KIR gene–gene model, which also examined the impact of KIR gene mismatching between recipient–donor pairs. However, no statistically significant association was discovered when overall mismatched KIR or aKIR genes were considered. As a result, the frequency of CR was first independently assessed in patients who had received livers from donors with any of the known specific HLA-I ligands for each considered KIR and who had inhibitory KIR2DL1+/KIR2DS1+ and KIR2DS4+ genes.

The authors compared transplants from C1-ligand-negative donors to those from KIR2DL2+/S2+ recipients and donors. A CR increase was noticed following transplants between KIR2DL3+ recipients and donors carrying the C1-ligand. The multivariable confirmed and strengthened this association. Other iKIR/ligand combinations, such as KIR3DL1 with its Bw4-ligands, were not connected to significant CR changes. Allograft survival decreased overall in patients suffering from CR compared to those who did not experience CR episodes. The authors observed that the differences were borderline significant at five years and significant at ten years. In other words, the probability of graft survival significantly increased at 5 and 10 years in transplants from donors with HLA-C1-ligands, compared to those in which the HLA-C1-ligand was absent. However, no differences were discovered when the impact of whether or not a donor carried HLA-Bw4 ligands was examined.

The findings of this study demonstrate the importance of KIR- and KIR ligand-dependent alloreactivity in late liver allograft outcomes, as each KIR+ cell is “encouraged” to sense the absence of a ligand. Due to the inflammatory environment created by increased cytotoxicity and NK or T cell activation, temporary liver allograft damage is encouraged. Additional studies on the allelic variations in KIR and HLA ligands, gene copy number variations and their levels of expression, and the high morbidity–mortality rates of patients in the early post-transplantation period are required to confirm these findings.

Finally, this Special Issue presents thought-provoking articles on the diverse responses of NK cells and related cells in the regulation, modulation, and influence of a series of pathologies, ranging from transplantation to oncological and pathological processes. NK cells are more active than ever as armed protectors of innate immunity; however, with their modulating role in acquired immunity, future studies will continue to discover relationships between these components of the immune response.
